# Evaluation of the pathogenicity of a rescued avian metapneumovirus subtype B strain in China

**DOI:** 10.3389/fvets.2025.1704092

**Published:** 2025-10-21

**Authors:** Zekun Yu, Chengyuan Jiang, Aili Guo, Haimin Wang, Chen Yuan, Tairan Sun, Qinye Song

**Affiliations:** ^1^College of Veterinary Medicine, Hebei Agricultural University, Baoding, China; ^2^Shandong Sinder Technology Co., Ltd., Weifang, China; ^3^Tangshan Zoo, Tangshan, China; ^4^Baoding Animal Disease Control and Prevention Center, Baoding, China

**Keywords:** avian metapneumovirus subtype B, virus rescue, phylogenetic analysis, pathogenicity, animal model

## Abstract

Avian metapneumovirus sub-type B (aMPV-B) is now widespread in China, causing significant declines in egg production among layer hens. However, the characteristics of sub-clinical infections and co-infections often resulted in low viral isolation rates, impeding research on its pathogenic mechanisms. To clarify the pathogenicity of Chinese aMPV-B field strains, we generated a strain B1 using reverse genetics and evaluated its pathogenicity in specific-pathogen-free (SPF) chickens. The complete 13,516 nt genome of strain B1 was assembled through segmented sequencing and alignment, exhibiting 96.5 to 98.7% sequence homology with prototype sub-type B strains, and > 98.6% identity with Chinese isolates. The rescued strain B1 was generated using a three-plasmid rescue system. In Vero cells, the rescued B1 induced characteristic syncytium formation, reaching the peak of the viral titer at 5 days post-infection (dpi). SPF chickens inoculated intranasally exhibited mild clinical signs dominated by nasal scratching and head shaking. The symptoms persisted for approximately 10 days, with the most severe at 5 days post-challenge (dpc). Oropharyngeal viral shedding peaked at 3 dpc and lasted around 7 days, and the predominant viral replication was in the upper respiratory tract, causing mucosal damage to nasal turbinates. Moreover, a challenge dose >10^2.0^ TCID_50_ elicited pronounced shedding peaks with similar shedding kinetics but low-dose viral challenge prolonged the viral incubation period. Under the 10^4.0^ TCID_50_ challenge dose, SPF chickens of different week-ages exhibited consistent virus shedding trends. This study advances the understanding of pathogenic features of Chinese aMPV-B strain and provides critical data for developing targeted control measures.

## 1 Introduction

Avian metapneumovirus (aMPV), a member of the genus metapneumovirus within the family *Pneumoviridae*, is an enveloped, single-stranded, negative-sense RNA virus that causes respiratory and reproductive diseases in poultry (1). Divided into four sub-types (A, B, C, and D) based on genetic and antigenic differences in the attachment glycoprotein (2), aMPV primarily infects turkeys and chickens, causing turkey rhinotracheitis (TRT), swollen head syndrome (SHS), and reductions in egg production. Sub-types A and B spread globally, while C and D have more limited geographic distribution (3–6). The virus targets the upper respiratory tract epithelium, leading to cilia loss, inflammation, and secondary bacterial infections, which intensify economic losses in poultry industries (7, 8).

The genome of sub-type B aMPV spans approximately 13.5 nt, encoding sequentially from the 3′-end: nucleocapsid protein (N), phosphoprotein (P), matrix protein (M), fusion protein (F), matrix protein 2 (M2), small hydrophobic protein (SH), attachment glycoprotein (G), and large polymerase protein (L). Notably, the M2 gene produces two distinct proteins (M2.1 and M2.2) through RNA editing mechanisms (9). The viral envelope contains four structural proteins (M, F, SH, and G), while the viral genomic RNA complexed with N, P, M2.1, and L proteins forms ribonucleoprotein complexes (RNPs). Crucially, naked viral RNA lacks infectivity, and RNPs serve as the fundamental functional units for viral transcription and replication.

Phylogenetic analyses show that aMPV-B strains cluster into a monophyletic group, with < 90% nucleotide identity in the G gene compared to other subtypes. Despite extensive studies in Europe, the molecular pathogenic mechanism of aMPV-B remains incompletely understood, particularly for Asian strains. In China, aMPV-B has been increasingly detected since its first identification in 1999. Serosurveys indicate widespread prevalence across multiple provinces, with flock-level seropositivity exceeding 50% in Shandong, Hebei, and Liaoning. The LN16 strain, isolated from broiler breeders with SHS in Liaoning in 2016, was the first fully sequenced Chinese aMPV-B isolate (10). Genomic analyses confirmed 97.6% nucleotide identity with European aMPV-B strains, highlighting its role in local outbreaks. Experimental infections show that aMPV-B induces respiratory lesions in chickens, with viral shedding peaking between 2 to 3 days post-infection (dpi) (11). However, virological data remain scarce due to challenges in viral isolation and the limited availability of Chinese aMPV-B isolates. This shortage not only impedes the progress of research on the pathogenic mechanism of aMPV-B, but also affects the development of region-specific diagnostic tools and vaccines.

To fill this gap, this study analyzed the genomic characteristics of a Chinese aMPV-B strain, investigated its pathogenicity and excretion shedding dynamics. This research will contribute to the study of the pathogenic mechanism of aMPV-B and the development of an animal model.

## 2 Materials and methods

### 2.1 Case history and sample collection

In March 2022, a flock of 160-day-old laying hens in Chifeng City, Inner Mongolia Autonomous Region, China, showed respiratory symptoms, including SHS, with no mortality. Nasal and oropharyngeal swabs, nasal turbinate, and lung samples were collected. Genotype identification of the samples was performed using the RT-PCR method described in the previous literature (10). And the case was confirmed to be caused by aMPV-B.

### 2.2 Viral genome sequencing and analysis

The viral RNA was extracted from the samples using TRIzol reagent following the manufacturer’s instructions. The full-length genome of the aMPV-B strain from the positive samples was obtained through overlapping RT-PCR amplification and subsequent sequence assembly, designated as strain B1. The resulting genome sequence of strain B1 was aligned with published aMPV genomes (Table 1) using MEGA 11 software, and phylogenetic trees were constructed based on the homologous alignment results.

**Table 1:** Information of the aMPV strain.

Virus strain	Subgroup	GenBank ID
aMPV-B_chicken_USA_ADRDL-6	B	PP273461
aMPV-B_turkey_USA_ADRDL-5	B	PP273460
aMPV-B_turkey_USA_SEP-RS1	B	PQ382890
aMPV-B_Brazil_1891_E2_19	B	OP572409
aMPV-B_turkey_France_VCO3_60,616	B	AB548428
aMPV-B_turkey_Hungary_657_4	B	MN729604
aMPV-B_chicken_Korea_SNU21004-E5	B	OR461285
aMPV-B_chicken_Korea_SNU21004-V12	B	OR461284
aMPV-B_chicken_Korea_21004-PLQ7	B	OM249787
aMPV-B_chicken_Korea_21004	B	OM249786
aMPV-B_chicken_Korea_SC1509	B	OR461286
aMPV-B_chicken_China_LN16	B	MH745147
aMPV-B_chicken_China_WH2022	B	OP036743
aMPV-A_chickern_Mexico_3155_22	A	ON854014
aMPV-A_turkey_USA_24–003048-001	A	PP442012
aMPV-A_chicken_Brazil_SP_669_2003	A	MF093139
aMPV-A_turkey_UK_LAH A	A	NC039231
aMPV-A_turkey_Italy_259-01 03	A	JF424833
aMPV-C_Mallard_Netherlands_1_2017	C	OM256458
aMPV-C_mallard_Netherlands_1_2019	C	OM179883
aMPV-C_duck_China_GDY	C	KC915036
aMPV-C_duck_China_2022_HL1	C	OR365551
aMPV-C_duck_China_2022_HF3	C	OR365552
aMPV-C_duck_China_CL	C	PP662686
aMPV-C_duck_France	C	HG934338
aMPV-C__turkey_USA_MN2a_97	C	FJ977568
aMPV-C_pheasant_Korea_PL-2	C	EF199771
aMPV-C_turkey_USA_Colorado	C	AY590688
aMPV-D_turkey_France_Fr85.1	D	HG934339

### 2.3 Virus rescue

**Figure 1:**
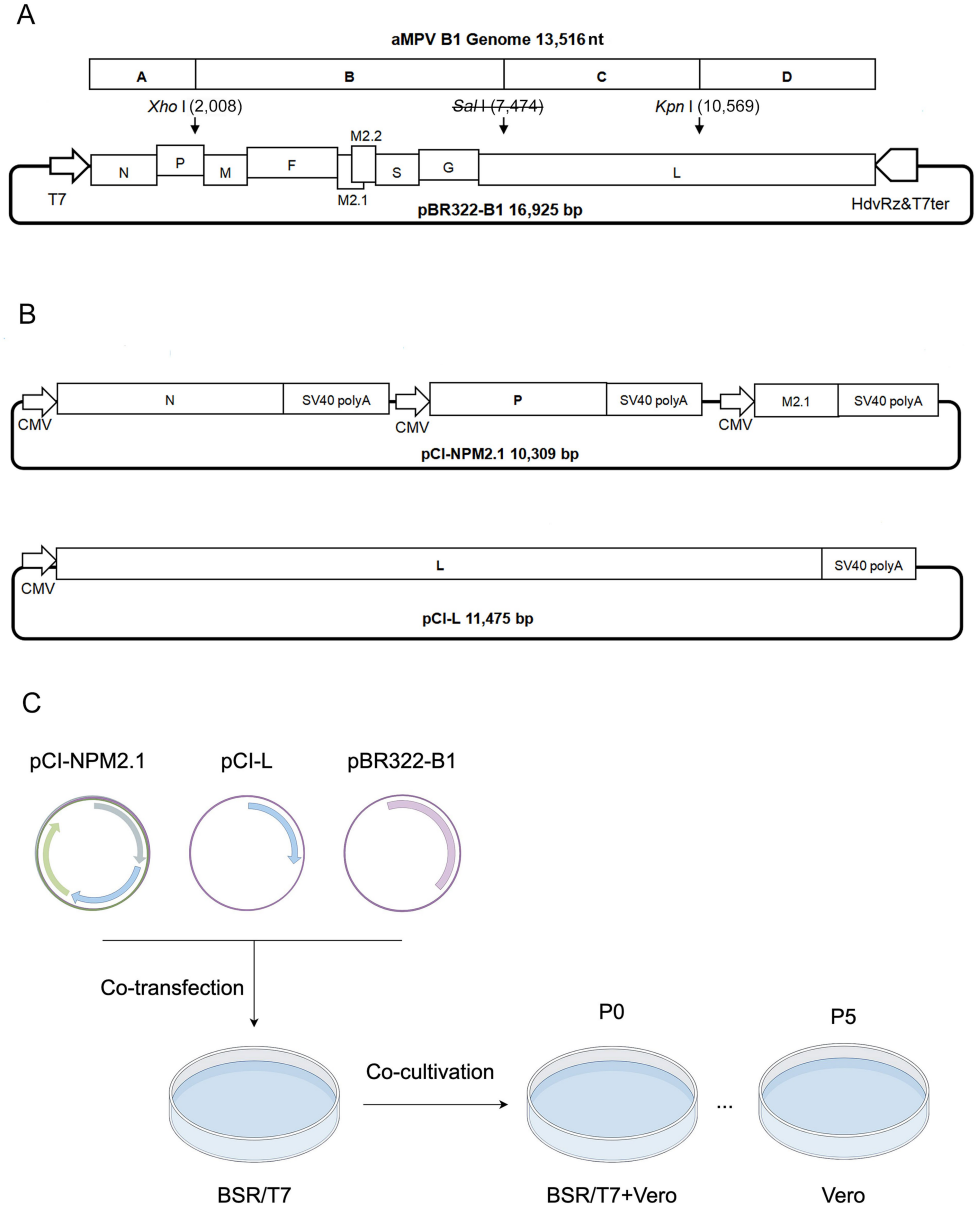
Schematic diagram of infectious clone construction and virus rescue. **(A)** Construction diagram of pBR322-B1 plasmid. A, B, C, and D indicate the fragments range to viral cDNA; T7: T7 promoter; HdvRz&T7ter: Hepatitis delta virus ribozyme and T7 terminator; *Sal* I: Genetic marker. The numbers in parentheses indicate the positions of restriction sites in the plasmid. **(B)** Diagram of helper plasmids pCI-NPM2.1 and pCI-L. **(C)** Schematic workflow for rescue of the strain B1.

The viral genome was divided into four fragments (A, B, C, and D) based on inherent restriction sites (*Xho* I, *Kpn* I) and cloned into the low-copy pBR322 vector to construct the genomic plasmid (Figure 1A). A silent mutation (U7,474C) in the viral genome was engineered to eliminate the *Sal* I restriction site, serving as a genetic marker. The open reading frames (ORFs) of three shorter genes (N, P, M2.1) were inserted into a single pCI-Neo expression plasmid, each under an independent expression cassette for autonomous transcription. The ORF of L gene was separately cloned into another pCI-Neo plasmid (Figure 1B). For details on primers and components, please refer to the Supplementary material. These two constructs served as helper plasmids for viral rescue. Co-transfection of the genomic plasmid and helper plasmids into BSR/T7 cells, followed by co-cultivation with Vero cells, generated the primary rescue virus (P0). For the viral infection, Vero monolayers with > 80% confluency in flasks were incubated with 5% (v/v) P0 virus suspension. After 2 h of adsorption, cells were overlaid with maintenance medium (2% fetal calf serum) for 6–7 d (P1). The culture was harvested and stored at −80 °C. Following freeze–thaw cycling, supernatants were passaged 5 times in Vero cells until distinct cytopathic effects (CPE) emerged (Figure 1C).

### 2.4 Indirect immunofluorescent assay (IFA)

The cells infected with rescued strain B1 were fixed using 4% neutral paraformaldehyde for 2 h at room temperature followed by washing 3 times with tris-buffered saline with tween-20 buffer (TBST). The fixed cells were blocked with 4% skim milk for 1 h. After three additional TBST washes, the cells were incubated with a 1:300 dilution of aMPV-B positive serum (Polyclonal antibodies were produced in SPF chickens following infection with the aMPV-B.) as the primary antibody for 1 h at room temperature. The cells were subjected to TBST wash, followed by incubation with a 1:300 dilution of Alexa Fluor 488 conjugated rabbit anti-Chicken IgG antibody (BBI LIFE SCIENCES, D110204) as the secondary antibody for 1 h in the dark. After TBST wash, the cells were mounted using a dihydrochloride (DAPI) solution and observed under a fluorescence inverted microscope (OLYMPUS, DP74).

### 2.5 Western-blotting

Vero cells infected with the rescued strain B1 were lysed using radio immunoprecipitation assay lysis buffer (RIPA, Solarbio, R0010). The supernatant was collected after centrifuging the cell lysate at 13,000 g for 5 min and mixed with an equal volume of 2 × SDS loading buffer, denatured at 100 °C for 10 min. Samples were electrophoresed on 12% tris-glycine polyacrylamide gels. Proteins were transferred to PVDF membranes (Solarbio, R0010) via semi-dry transfer apparatus. Membranes were blocked with 4% skim milk for 1 h, probed with mouse anti-aMPV-B N protein antiserum (1:500 dilution, the polyclonal antibodies were obtained from mice immunized with the truncated N protein of aMPV-B) for 1 h, and washed thrice with TBST. After incubation with HRP-conjugated goat anti-mouse IgG secondary antibody (1:5,000 dilution, TransGen Biotech, HS201-01) for 1 h at room temperature, membranes were washed and developed with DAB substrate for band visualization.

### 2.6 RT-PCR-RFLP

Genetic marker verification was performed using *Sal* I diagnostic primers: F: 5′-ACCAGGTAG GAACTTACAATCCTAG-3′, R: 5′-AGTAGCTTTATAATCTTGAGCACTC-3′ (amplicon size: 1,331 bp). The target nucleic acid fragment containing the genetic marker was amplified by PCR, digested with *Sal* I restriction endonuclease, and differentiated from the nucleic acid from positive sample by agarose gel electrophoresis.

### 2.7 Virus titer and growth curve plotting

To determine the growth kinetics of the rescued strain B1, cell monolayers were infected at a multiplicity of infection (MOI) of 0.1 and incubated for 6 days. The culture supernatants were collected every 24 h. The 50% tissue culture infectious dose (TCID_50_) of each collected sample was determined based on CPE and IFA using the reed-muench method, and the virus growth curve was plotted using TCID_50_ values at different time points.

### 2.8 Animal experiments

**Figure 2:**
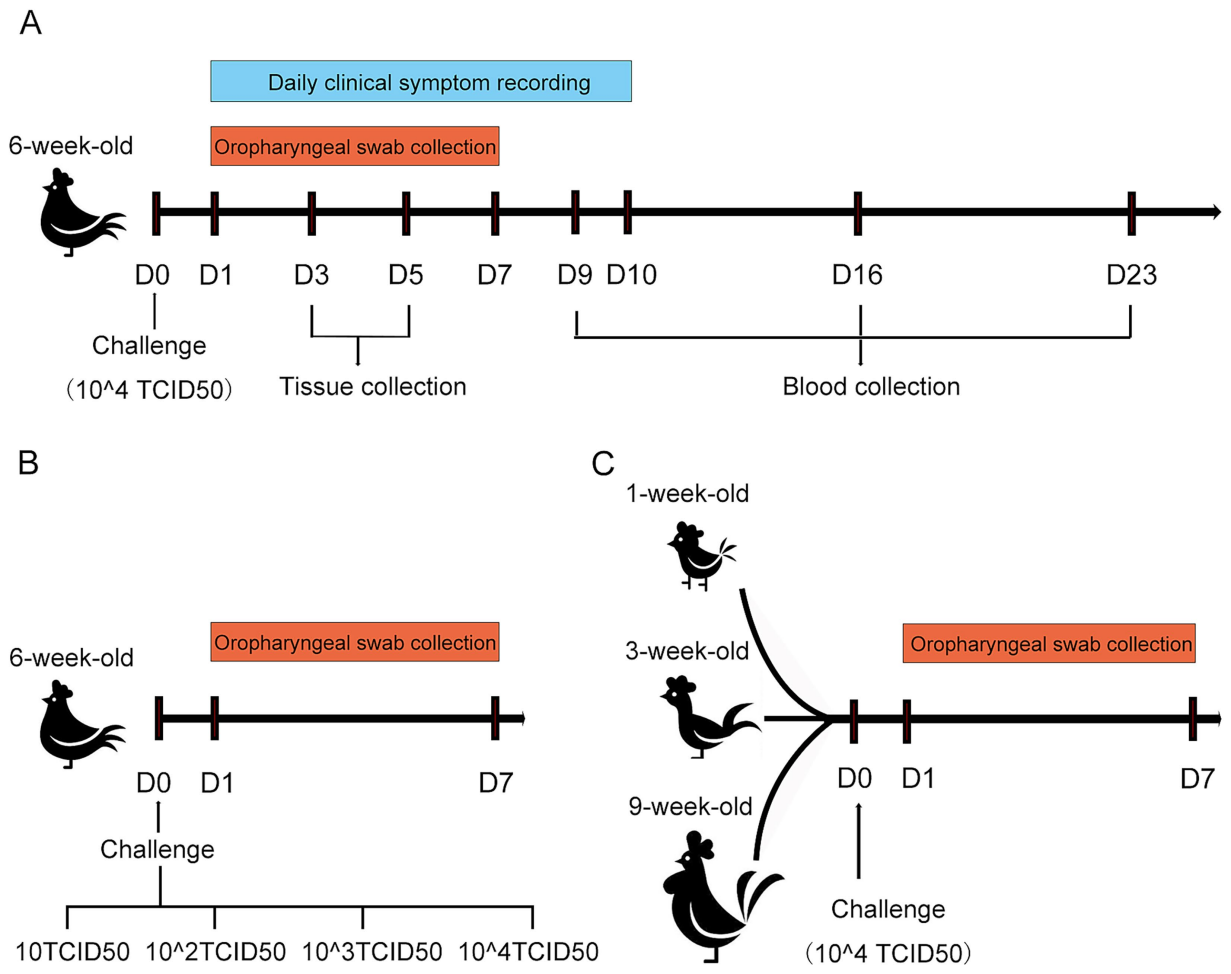
Schematic diagram of the SPF chicken challenge experiment for rescued strain B1. **(A)** Schematic diagram of the challenge assay in 6-week-old SPF chickens. **(B)** Schematic diagram of challenge under different doses in 6-week-old SPF chickens. **(C)** Schematic diagram of challenge in SPF chickens at different weeks of age.

To characterize the pathogenicity of the rescued strain B1, 6-week-old SPF chickens purchased from Beijing Boehringer Ingelheim were randomly assigned to two groups, with 25 chickens in each group. In Group 1, each chicken was inoculated intranasally with 2 × 10^4.0^ TCID_50_ of the P5 generation of the rescued virus. Group 2 served as a control and received the same volume of PBS. After challenge, clinical symptoms and morbidity rates were recorded daily. Clinical symptoms were scored as follows: asymptomatic (scored as 0 points), head shaking and nasal scratching (scored as 1 points), and head swelling and nasal discharge (scored as 2 points). A morbidity curve was generated based on daily scores. Oropharyngeal swabs were collected from both groups on days 1 to 7 post-challenge, put into PBS (pH 7.4) and vortexed thoroughly for viral RNA extraction and viral shedding quantification. Nasal turbinate, trachea, and lung tissues were harvested on days 3 and 5 post-challenge. The parts of these tissues were fixed in 4% neutral paraformaldehyde for hematoxylin and eosin staining (HE) and pathological observation, and the others were pulverized in liquid nitrogen, and 0.1 g was used to extract total RNA for viral load measurement in each tissue. Blood samples were collected from both the challenge group and the control group at 9, 16, and 23 days post-challenge (dpc) for serological testing (Figure 2A).

To determine the effect of challenge dose on viral shedding, a total of seventy-five 6-week-old SPF chickens were randomly allocated into five groups (15 chickens per group). The P5 generation of the rescued strain B1 was serially diluted in PBS to four titer levels: 10 TCID_50_, 10^2.0^ TCID_50_, 10^3.0^ TCID_50_, and 10^4.0^ TCID_50_. Each group was challenged with 200 μL of the virus via intranasal. The remaining group served as an unchallenged control. Oropharyngeal swabs were collected from all experimental chickens at 1–7 dpc, and viral shedding levels were quantified by real-time RT-PCR (RT-qPCR) (Figure 2B).

To investigate whether viral shedding dynamics was significantly affected by host age, thirty SPF chickens each at ages 1-, 3-, and 9-week-old were utilized, respectively. Thirty birds at various ages were randomly allocated into two groups. The challenge group was inoculated with 200 μL of 10^4.0^ TCID_50_ rescued B1 (P5) via intranasal instillation, while the control group remained unmanipulated. Oropharyngeal swabs were collected from all experimental chickens at 1–7 dpc, and viral shedding levels were quantified by RT-qPCR (Figure 2C).

### 2.9 Quantitative RT-PCR detection of virus

Oropharyngeal swabs were used for viral RNA extraction. Viral RNA was quantified using one-step RT-qPCR. The forward primer (5′-AATAGTCCTCAAGCAAGTCCTCAGA-3′), reverse primer (5′-TGTTGTAATTTGACCTGTTCTATACT-3′), and probe (FAM-CTGGTGTTATCAGCCTTAG GCTTGACGCT-BHQ) were employed to amplify a target of the G gene. The viral RNA copy number was calculated from the CT values of the samples.

### 2.10 ELISA detection of specific antibody

Blood was drawn from the jugular vein of chickens and incubated at 37 °C for 1 h to allow coagulation. The coagulated blood underwent centrifugation at 3,000 rpm for 15 min, and the upper serum layer was harvested. The antibody titer was determined using the ELISA method (avian pneumovirus antibody test kit, No. 99–44,300 in China). Experimental procedures were performed following the manufacturer’s instructions and the absorbance was measured using a photometer (ThermoFisher, Multiskan™ FC Microplate) at 650 nm. An S/P ratio > 0.20 was considered positive, and antibody titer = 10^[1.09(log10(S/P)) + 3.36]^.

### 2.11 Serum neutralization test

The isolated serum was heat-inactivated at 56 °C in a water bath. A two-fold serial dilution of the serum (starting at 1:2) was prepared and mixed with an equal volume of virus solution containing 200 TCID_50_ of the rescued strain B1. The mixture was incubated at 37 °C for 2 h following being added to Vero cell monolayers in a 96-well plate, seven replicates conducted for each dilution of serum. After a 2 h incubation at 37 °C, the supernatant was replaced with a 2% FBS maintenance medium. The cells were further cultured for 7 days, and the antibody neutralization titer was calculated using the reed-muench method.

## 3 Results

### 3.1 Phylogenetic and genome analysis of strain B1

**Figure 3:**
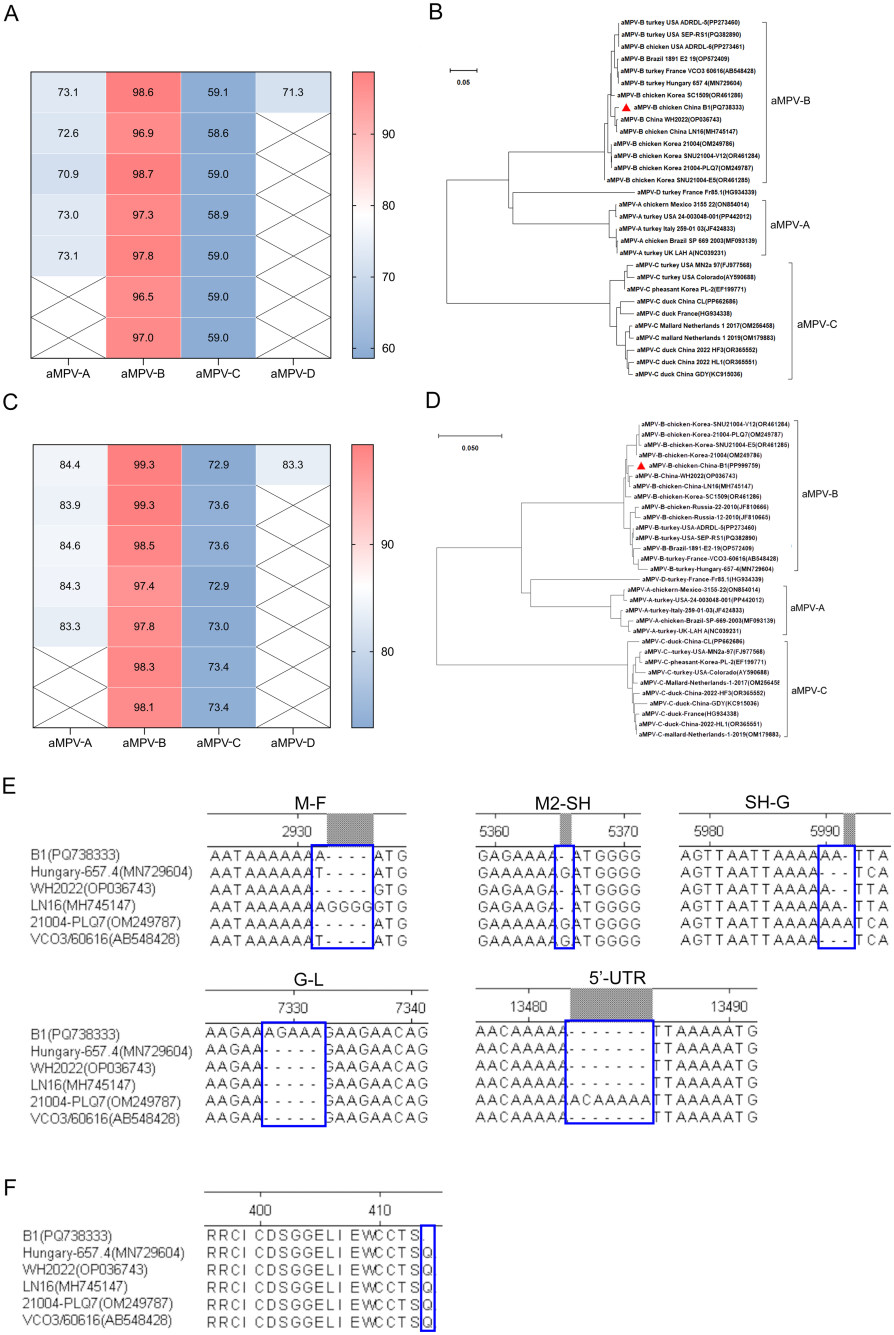
Sequence homology and phylogenetic analysis. **(A)** Genomic sequence homology analysis. This analysis demonstrated the degree of similarity between the B1 strain and other aMPV strains. **(B)** Phylogenetic tree (neighbor-joining method). This phylogenetic tree, constructed based on aMPV genome sequences, the strain B1 marked by a red triangle. **(C)** F protein sequence homology analysis. This analysis highlights the sequence homology of the B1 F protein with other aMPV strains. **(D)** Phylogenetic tree (neighbor-joining method) of F protein, based on aa sequence of aMPV, the F protein of strain B1 denoted by a red triangle. **(E)** INDELs in the intergenic regions between GE and GS of aMPV-B genome sequences. **(F)** The comparison result of aa residue of G protein at carboxyl terminal.

After confirming that the case was caused by aMPV-B, the complete genome sequence of the virus named strain B1 (GenBank accession number PQ738333) was obtained through segmented amplification and assembly. Phylogenetic analysis showed that the strain B1 clustered within the same branch as the earliest reported European sub-type B isolate, VCO3/60616, with 97.3% nucleotide homology. Compared to other published sub-type B strains, the strain B1 had homology levels above 96%, while homology with sub-type A, C, and D strains ranged from 56 to 73%. Notably, the strain B1 showed greater similarity with the Chinese strains LN16 and WH2022, at 98.7 and 98.6% respectively, exceeding that of American and European sub-type B strains (Figure 3A; Table 2). Phylogenetic analysis further demonstrated that the strain B1 along with two Chinese strains LN16 and WH2022 formed a distinct sub-clade, separating clearly from European and American strains (Figure 3B). Based on the F protein, the strain B1 had 98.1% homology with the VCO3/60616 strain and 99.3% with both LN16 and WH2022 strains, these predominant strains in China belonged to the same sub-lineage (Figures 3C,D; Table 2).

**Table 2:** Homology comparison between strain B1 and representative aMPV strains.

Virus strain	Sub-type	Country	GenBank ID	Genome (nucleic acid)	F protein (aa)
VCO3_60,616	B	France	AB548428	97.3%	98.1%
LN16	B	China	MH745147	98.7%	99.3%
WH2022	B	China	OP036743	98.6%	99.3%
ADRDL-6	B	USA	PP273461	97.1%	98.2%
LAH A	A	UK	NC039231	73.9%	84.2%
Colorado	C	USA	AY590688	61.5%	73.0%
GDY	C	China	KC915036	61.8%	72.8%
Fr85.1	D	France	HG934339	73.9%	83.3%

The B1 genome spanned 13,516 nt, encoding eight proteins (N, P, M, F, M2, SH, G, and L) from the 3’ end, which was consistent with the genomic structure of other metapneumoviruses. Sequence alignment showed that while the coding regions (CDSs) of sub-type B strains were largely conserved, the strain B1 exhibited genome length variations due to insertions or deletions (INDELs) in the intergenic regions between gene end (GE) and gene start (GS) (Figure 3E). Although the CDS regions of the viral proteins were identical across all strains, the strain B1 harbored a C1,240U transition in the G gene. This mutation introduced a premature termination codon, causing truncation of the carboxyl terminus with loss of a glutamine residue (Figure 3F). Critical virulence sites within the strain B1 F protein which served as the primary virulence determinant for aMPV-B were conserved relative to published field strains, confirming its wild-type pathogenic features (Table 3).

**Table 3:** Amino acid residue comparison in F proteins of virulent and Vero cell-passage attenuated strains.

Site		Virulent strain		Attenuated strain	
	B1	LN16-V	VC03/60616	LN16-A	VC03/50
89	I	I	I	I	F
323	E	E	E	K	-
396	K	K	K	R	-
522	L	L	S	P	-

### 3.2 Cultivation characteristics of rescued strain B1

**Figure 4:**
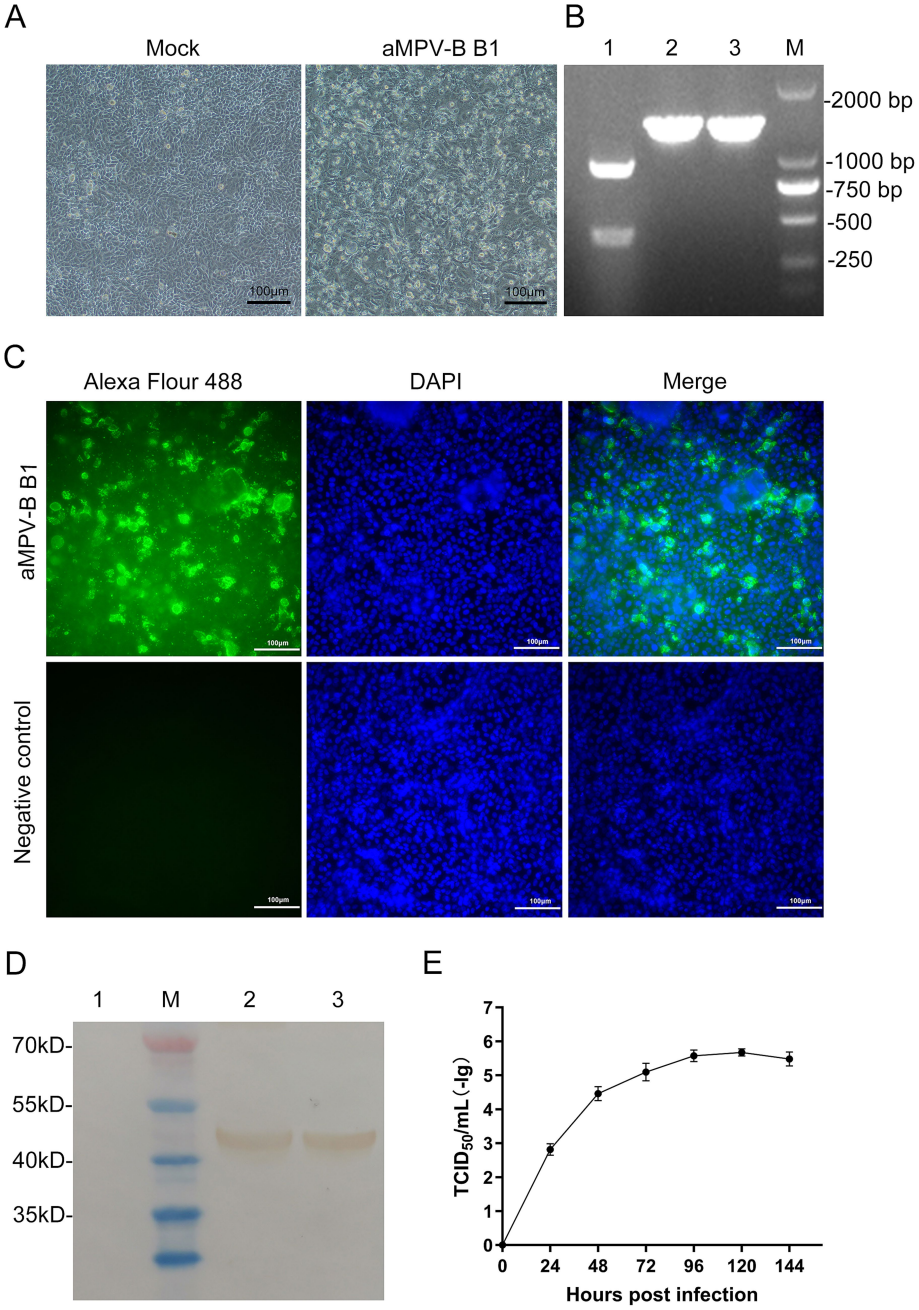
Identification and Growth kinetics of rescued strain B1. **(A)** CPE were observed in Vero cells infected with the rescued strain B1 at the 5th passage. (Bars, 100 μm). **(B)** Results of RT-PCR-RFLP analysis. Lane 1: Nucleic acid from positive sample, Lanes 2 and 3: Nucleic acid of rescued strain B1 cultured in Vero cells, Lane M: DL2,000 DNA marker. **(C)** Vero cells at 96 h post-infection were incubated with a chicken antibody against aMPV-B (Bars, 100 μm). **(D)** Results of western-blotting analysis. Lane M: Protein marker. Lane 1: Vero cells control. Lanes 2 and 3: Vero cells incubated with rescued strain B1. **(E)** Growth kinetics of the aMPV-B strain B1 in cultured cells. Vero cells were infected with rescued strain B1 at 0.1 MOI and harvested at the indicated time points for viral titration. Data are presented as mean ± standard deviation (*n* = 3).

Following 5 serial blind passages, infected Vero cells developed typical CPE, including cell rounding, elongation, increased intercellular gaps, detachment and syncytium formation. Control cells showed no significant changes (Figure 4A). PCR amplification of the positive nucleic acids from clinical specimens yielded a DNA fragment containing the *Sal* I restriction site. Digestion with *Sal* I generated cleavage segments at 910 bp and 400 bp, whereas the rescued B1 amplicon (1,310 bp) remained intact showing a single band due to engineered *Sal* I site elimination (Figure 4B).

To clarify the infection characteristics of the virus, IFA was performed using aMPV-B positive serum. The distinct fluorescence emitted in infected Vero cells, whereas no fluorescence was observed in the control group (Figure 4C). In addition, western-blotting analysis of infected cells detected a 44 kDa band corresponding to the viral N protein, with no specific reactivity observed in mock-infected controls (Figure 4D). According to the viral growth curve, the rescued B1reached the plateau phase at approximately 120 h post-inoculation in Vero cells, with a peak titer of 10^-5.5^/mL (Figure 4E).

### 3.3 Mild clinical signs and pathological changes in 6-week-old chickens

**Figure 5:**
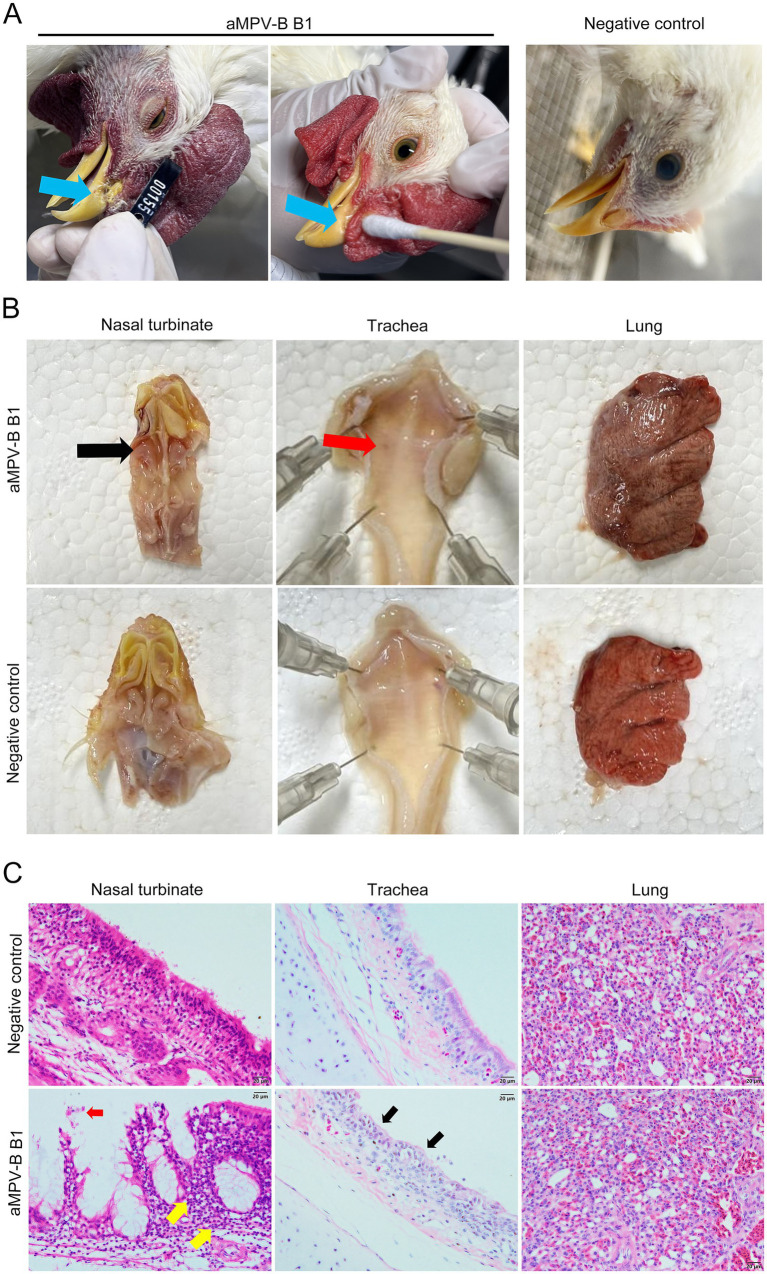
Pathogenic analysis of rescued strain B1 in 6-week-old SPF Chickens. **(A)** Nasal discharge in chickens after challenge. Blue arrows indicated strain-associated clinical signs included increased nasal secretion, serous nasal discharge, and yellowish crusts on nasal part. **(B)** Gross lesions of the respiratory tract. Black arrow indicated mucosal congestion in the nasal turbinate, red arrow pointed to tracheal bleeding spots. **(C)** Histopathological changes in the nasal turbinate, trachea, and lung of infected chickens. Red arrow indicated desquamation of the nasal turbinate mucosa; yellow arrow demonstrated inflammatory cell infiltration. Black arrow pointed to hyperplasia of tracheal mucosal epithelium with deciliation, (Bars, 20 μm).

**Figure 6:**
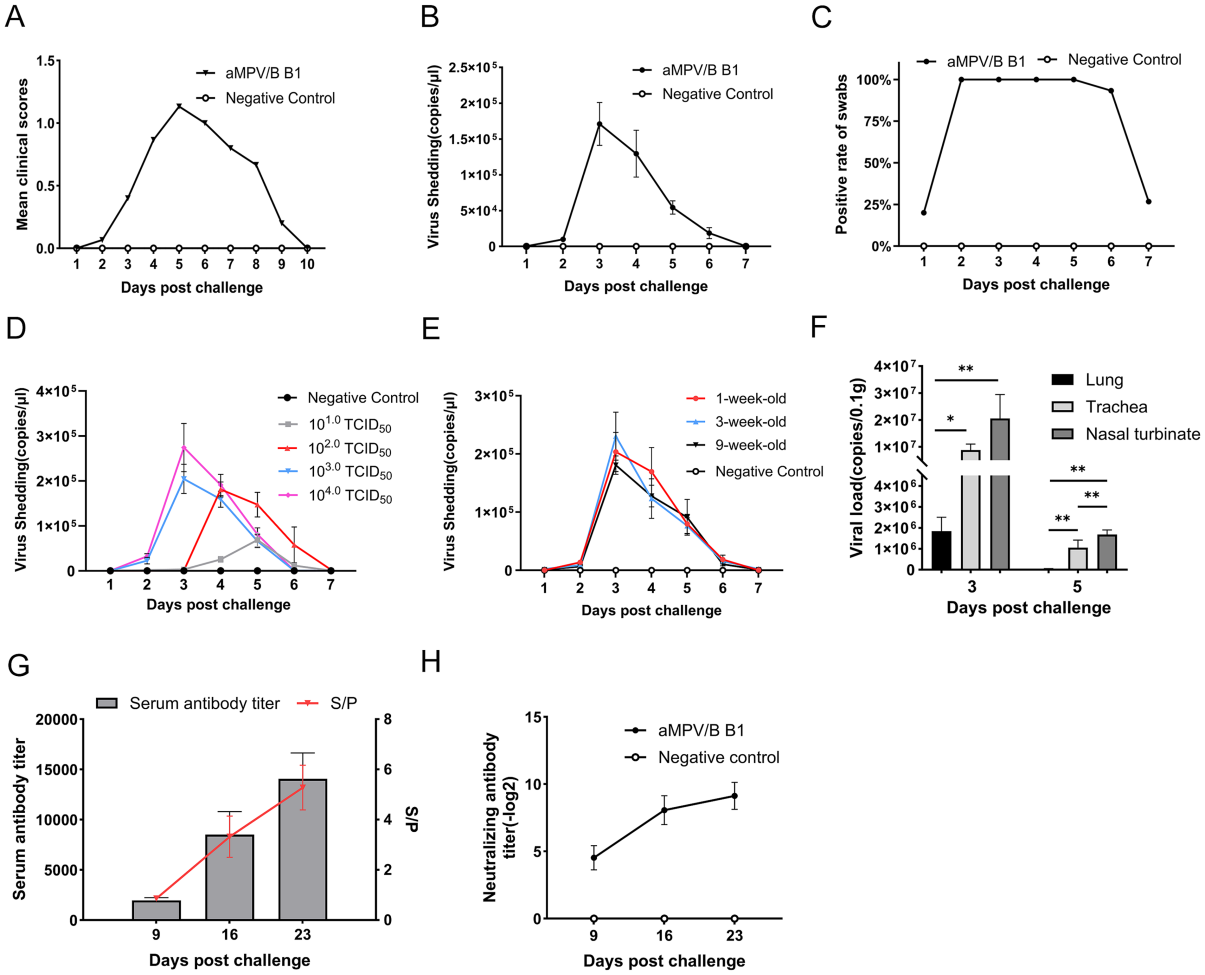
Oropharyngeal virus shedding, tissue viral loads and serological analysis in challenged chickens. **(A)** Mean daily clinical scores after rescued B1 challenge in chickens (*n* = 15). **(B)** Quantification of shedding in oropharyngeal swabs from rescued strain B1-challenged chickens using RT-qPCR, error bars indicate ± SE (*n* = 15). **(C)** Positive rate of nucleic acid in oropharyngeal swabs of rescued strain B1-challenged chickens (*n* = 15). **(D)** Viral shedding kinetics in 6-week-old SPF chickens challenged with 10 to 10^4.0^ TCID_50_ doses, error bars indicate ± SE (*n* = 15). **(E)** Viral shedding curve from 1-,3-,9-week-old SPF chickens challenged with 10^4.0^ TCID_50_ dose, error bars indicate ± SE (*n* = 15). **(F)** Viral loads of rescued strain B1 in tissues of challenged chickens (*n* = 5, statistical significance was determined by two-way ANOVA, *p ≤ 0.05, **p ≤ 0.01). **(G)** Antibody titers in sera from experimentally infected chickens. The S/*p* values (right ordinate) and serum antibody titers (left ordinate) are shown, error bars indicate ± SD (*n* = 10). **(H)** Neutralizing antibody titers in infected chickens, evaluated based on viral load at 100 TCID_50_, error bars indicate ± SD (*n* = 5).

Following the challenge, 6-week-old SPF chickens developed mild respiratory symptoms, including lethargy, huddling, frequent head shaking, and nasal scratching. Some showed slight swollen eyelids and nasal mucus discharge (Figure 5A). The disease course lasted approximately 8 to 10 d, with symptoms peaking on day 5 post-challenge. At this time, 86.7% exhibited head shaking and nasal scratching, while 26.7% had swollen eyelids and increased nasal mucus. The average disease index was 1.13, and no symptoms were observed in the control group (Figure 6A). Necropsy analysis revealed nasal turbinate mucosal hyperemia with mucoid exudate in challenged chickens compared with the control group. Additionally, petechial hemorrhages were observed in the trachea of infectious chickens, whereas no significant pathological alterations were found in pulmonary tissues (Figure 5B). Histopathological examination showed that the nasal turbinate mucosa of challenged chickens had cilia and epithelial cell loss, mucosal structural disruption, and heterophilic cell infiltration. In contrast, the control group had tightly arranged pseudostratified columnar epithelium and scattered tubular glands in the lamina propria and submucosa. The tracheal mucosa of challenged chickens showed irregular epithelial cell proliferation and cilia loss, while the control group had a compact epithelial arrangement and intact cilia. In addition, compared with the control chickens, no significant pathologic alterations were observed in the lungs of challenged chickens (Figure 5C).

### 3.4 The peak of viral shedding varied with challenge dosages

To analyze viral shedding, oropharyngeal swabs of 6-week-old SPF were collected from days 1 to 7 post-challenge at a dose of 10^4.0^ TCID_50_ and the viral loads in samples were quantified by RT-qPCR. The rescued strain B1 was shed for approximately 1 week, peaking at day 3, followed by a gradual decline (Figure 6B). The positive rate for swabs collected on days 2 to 5 was 100%, while no shedding was detected in the control group (Figure 6C).

Moreover, the rescued strain B1 challenge in 6-week-old SPF chickens demonstrated dose-dependent oropharyngeal viral shedding kinetics, characterized by progressive attenuation of peak titers and temporal shifts with decreasing inoculum. Specifically, groups challenged with higher doses (10^4.0^ and 10^3.0^ TCID_50_) exhibited shedding profiles concordant with previous observations, achieving maximal viral shedding at 3 dpc followed by sustained decline over a 4 to 7 d shedding duration. However, the dosage of 10^4.0^ TCID_50_ cohort generated elevated viral shedding titers relative to the other dosage groups. Although the intermediate-dose cohort (10^2.0^ TCID_50_) displayed comparable kinetic patterns, peak shedding was delayed to 4 dpc with a marginal extension of shedding duration to approximately 7 days. Conversely, the lowest group (10 TCID_50_) failed to establish a discernible shedding peak (Figure 6D).

In addition, SPF chickens aged 1-, 3-, and 9- week, developed mild respiratory signs post-challenge, exhibited shedding kinetics identical to 6-week-old chickens under equivalent viral doses. The shedding cycle consistently spanned 7 days, in which days 1 to 2 represented the pre-shedding phase (latent period), day 3 marked peak viral excretion, and days 4 to 7 showed progressive decline to undetectable levels (Figure 6E).

### 3.5 There was a higher viral load in the nasal turbinate mucosa

The viral loads in various tissues were analyzed using RT-qPCR. The virus was mainly detected in the nasal turbinate, trachea, and lungs. Viral loads in respiratory tissues were significantly higher on 3 d than on 5 d, with clearance kinetics mirroring the viral shedding dynamics observed in oropharyngeal swabs. During the viral shedding period, viral loads in nasal turbinate mucosa were significantly higher than those in tracheal and pulmonary tissues (Figure 6F).

### 3.6 The rescued strain B1 induced specific antibodies

Seroconversion reached 100% by day 9 post-challenge, with S/P ratios increasing over the next two weeks. By day 9, 16, and 23, the mean antibody titer were 1:1,952.69, 1:7,968.32, and 1:12,950.01, respectively, (Figure 6G). Neutralizing antibody titer (Log2) followed a similar trend, reaching 4.53, 8.07, and 9.13, respectively. No antibodies were detected in the control group (Figure 6H).

## 4 Discussion

Sub-type B avian metapneumovirus has become widely prevalent globally, attracting increasing attention in China in recent years. Su reported that three subtypes (A, B, and C) of aMPV in a similar positive rate were detected in multiple regions of China using a triplex RT-qPCR method (12). However, Wang found that aMPV-B predominated in chicken infections (13). It is worth nothing that the detection rate of sub-type B strains is high in the flocks exhibiting SHS, the viral isolation rate has been very low. So far, only one strain LN16 in China, has undergone complete genome sequencing and systematic challenge experiments. To enhance our understanding of aMPV-B strains in China, we conducted full genome sequencing of the strain B1 obtained from a clinical case, clarified its culture characteristics, and elucidated its pathogenic features through challenge experiments in chickens.

Obtaining clinical samples with sole aMPV-B infections (without co-infection with other pathogens) is extremely challenging, which impedes reliable viral isolation. To circumvent limitations associated with suboptimal sample quality, this study employed a reverse genetics system to rescue a continuously passagable aMPV-B strain directly from the field specimens and the genomic sequence of the rescued virus strain faithfully represented the original sequence. Previously established aMPV rescue systems utilized a five-plasmid method, comprising the full-length genomic plasmid alongside four helper plasmids individually expressing N, P, M2.1, and L proteins (14–16). In this study, we reported a refinement of this approach by consolidating the three smaller genes (N, P, M2.1) into a single plasmid, thereby reducing the total plasmid count to three. This reduction enhanced viral rescue efficiency, as the probability of a transfected cell receiving three plasmids exceeds that of simultaneously incorporating five plasmids, consequently increasing the number of cells capable of viral packaging and thus the initial yield of viral particles. This refinement aligns with previous reports for influenza virus and Newcastle disease virus rescue systems, where reducing transfection plasmid counts similarly augmented rescue efficiency (17, 18).

As serial passage of aMPV-B in Vero cells could inherently introduce genomic mutations, restricting cell culture passages to lower generations minimizes genetic drift and preserves the wild-type characteristics of the viral strain (19), implying conducting viral infection experiments utilizing P5-generation virus stock was reasonable in this study. The lysine residue at position 369 of the F protein is a critical determinant of viral virulence. Mutation at this site impairs viral binding affinity to host cell receptors, thereby attenuating the viral replication efficiency. In strain B1, Lys369 was conserved, identical to reference field strains LN16 and VCO/60616, confirming its wild-type pathogenic characteristics.

Phylogenetic analysis of the viral genome revealed that strain B1 shares over 96% homology with published subtype B strains, indicating relatively global conservation of these strains, which aligns with prior findings on Chinese isolates (10). The F protein as a major neutralizing antigen of aMPV exhibited over 97.4% amino acid (aa) homology, exceeding genome-wide homology across strains (20, 21). This suggests that subtype B aMPV has not faced significant vaccine-induced selection pressure, and its major antigenicity remains stable, with a low overall mutation rate, similar to epidemiological trends in European strains (22, 23). Meanwhile, since the F protein primarily determines host tropism (24), its stability restricts the host range of subtype B aMPV infections, such as chickens and turkeys. Although sub-type B aMPV is generally conservative, the prevalent strains in China exhibit higher genetic homology. Additionally, specific mutations unique to Chinese isolates were identified, such as Pro and His at positions 82 and 87 of the SH protein, contrasting with Ser and Tyr in non-Chinese strains. Moreover, codon usage frequencies at many synonymous mutation sites in Chinese strains tend to align, differing from those in non-Chinese strains, which suggests that Chinese isolates may have originated from a common ancestor and evolved independently. Meanwhile, vaccines based on European strains could be considered for controlling aMPV-B in China based on the results of phylogenetic analysis; however, it remains necessary to develop vaccines targeting local strains.

Furthermore, a glutamine deletion did not significantly inhibit viral proliferation *in vitro*, suggesting that integrity of the G protein did not critically affect the infectivity of the virus. Previous studies indicated that G protein deletion, truncation, or substitution had no impact on in vitro viral replication but reduced virulence for aMPV subtypes (19, 21, 25, 26). Further research is needed to confirm the impact of the deletion of a glutamine in the G protein of the B1 strain on its virulence.

Sub-type B aMPV exhibits weak pathogenicity, with a low mortality in infections. The challenge experiments with rescued strain B1 revealed similar characteristics, with most SPF chickens showing only brief mild respiratory symptoms and no mortality. Clinical symptoms peaked 2 to 3 days after peak shedding of the virus, meaning the most pronounced symptoms occurred during the late shedding phase, which makes optimal time for sample collection complicated, thereby hindering pathogen isolation. The increasing trends of ELISA and neutralizing antibody detection results were consistent, indicating that the ELISA method can be utilized for monitoring and evaluating vaccine efficacy during development. This contributes to a quick assessment of vaccine efficacy. Tissue viral load analysis showed the virus primarily localized in the respiratory tract, with the highest titers in the turbinate, which was similar to the results of this study (27). The mild pathogenic features of sub-type B aMPV were reflected in its limited tissue damage, and the pathological changes mainly involved mucosal damage and inflammatory cell infiltration in the turbinate. In contrast, sub-type C strains caused more extensive pathological damage, with severe tissue destruction, hemorrhage, and inflammatory responses in the turbinate, trachea, lungs, and thymus in chicken and duck challenge experiments (27–29). The findings strongly suggest that, whether in clinical testing or the application of animal models, nasal turbinate tissues and oropharyngeal swabs are the preferred sampling sites, as they more faithfully represent the viral replication process.

For practical monitoring, oropharyngeal swab-based viral shedding detection allows scalable sample size expansion and longitudinal sampling, serving as the principal metric for staging disease progression. The multiple rounds of challenge experiments demonstrated that the viral shedding dynamics in SPF chickens were solely dependent on the challenge dose, with no significant influence from the age factor of the birds. In animal model applications, employing a high-titer challenge dose facilitates the collection of swab or nasal turbinate tissue samples during the peak shedding period (3–5 days post-inoculation). By quantifying the viral load, the infection progression can be indirectly inferred, thereby overcoming the limitation of evaluating aMPV-B pathogenesis due to its lack of distinct clinical manifestations. Moreover, as vaccine development often involves challenge trials in chickens of varying ages, these findings supported the adoption of a standardized sampling protocol regardless of age differences. This approach simplifies sample collection criteria and eliminates the need for age-specific adjustments in sampling strategies.

This study analyzed the genomic sequence and genetic characteristics of a prevalent strain of aMPV-B, characterized the replication and pathogenicity of the rescued aMPV-B strain B1 through viral culture, genome sequencing, and SPF chicken experiments. However, the limitation of the animal experiments lies in the fact that the secondary infections were not replicated in a natural setting, which prevented the occurrence of typical clinical symptoms such as the swollen head syndrome. In order to further investigate the pathogenic mechanism of the B1 strain, we will simulate the natural infection scenario and explore the animal infection model under the condition of secondary infection.

## 5 Conclusion

This study revealed that the molecular characteristics of the aMPV-B genome from a layer hen farm in China, successfully generated the rescued strain B1, demonstrated that the rescued virus induced mild upper respiratory tract symptoms with rhythmic viral shedding in chickens, found that oropharyngeal virus shedding duration was dose-dependent but age-independent in challenged birds, enriching the molecular characterization of aMPV-B and providing a foundation for animal model and vaccine development.

## Data Availability

The datasets presented in this study can be found in online repositories. The names of the repository/repositories and accession number(s) can be found in the article/Supplementary material.
