# Supplementary material for: Evaluation of the pathogenicity of a rescued avian metapneumovirus subtype B strain in China
Source: Front Vet Sci. 2025 Oct 21;12:1704092. doi: 10.3389/fvets.2025.1704092 (PMC12586556; doi:10.3389/fvets.2025.1704092)
Supplement: Supplementary file 1 [file Table_1.DOCX]

**SUPPLEMENTARY**

1 Viral genome sequencing primers

**Table S1. The primers for viral genome sequence**

| Primer Names | Sequence 5'→3' | Amplification  Length (bp) |
| --- | --- | --- |
| 3' RACE primer | CTCTGTGCATCTTCTTCCT | ~1,900 |
| 5' RACE primer | CCCGTCACGGAGTACCTC | ~1,700 |
| F2F | AGGACAATGCACCATCATC | 2,409 |
| F2R | CTCACACCTTCATAACATGATAC |  |
| F3F | CTGTGATACTGCAGCAGG | 2,417 |
| F3R | GCATATCACCATCAACAACTTG |  |
| F4F | TAGGACTGGTGTTATCAGC | 2,524 |
| F4R | GGTTCTTTATTCTTGGCCAC |  |
| F5F | ACAGGAAGAGTGCTCAAG | 2,382 |
| F5R | GCGCTTTGATAATTGACAAC |  |
| F6F | TAACATGATAGCAGGTACC | 1,977 |
| F6R | TCTTTTAGGCTCCTGTAGAC |  |

2 Primers required for constructing genomic plasmids and helper plasmids

**Table S2. The primers of plasmid constraction and fragment length**

| Primer Names | Sequence 5'→3' | Amplification Length (bp) |
| --- | --- | --- |
| FAF | ACGACTCACTATAGGGACGAGAAAAAAACGCATTCAAGTCACAATAG | 2,405 |
| FAR | TGCCTCAGATTCACCGCTCGAG |  |
| FBF | AGGATCAAAGCTCGAGCGGTGAATC | 5,485 |
| FBR | TATGGTCGGCCTATAATGCAAGACCCAATTGC |  |
| FCF | TATAGGCCGACCATACCTAAAGGATGAC | 3,118 |
| FCR | ATTTGTAATAGATTTGGTACCTGCTATC |  |
| FDF | GGTGAATCTGAGGCAGTAGTTAACATGATAGCAGGTAC | 2,987 |
| FDR | GATGCCATGCCGACCCACGGCAAAAAAACCGTATTCAATAC |  |
| LF | GGCTAGCCTCGAGAATTCGCCACCATGGACCCATCCAGTGAGC | 6,057 |
| LR | GCGGCCGCCCGGGTCGACCTATTTTGTGCTCAGTATGTACCCTGT |  |
| NPM2.1-NF | ATAGCGATAAGGATCTAGTTCATAGCCCATATATGGAGTTCCGC | 2,380 |
| NPM2.1-NR | ATAATCAAGTAGAGGTTTTACTTGCTTTAAAAAACCTCC |  |
| NPM2.1-PF | CCTCTACTTGATTATTGACTAGTTATTAATAGTAATCAATTACGGGGTCA | 2,149 |
| NPM2.1-PR | CACCATACGCGGATCGGTGCGGGCCTC |  |

Note: The underlined part represents the homologous sequence.

3 Sequences of transcriptional promoter, ribozyme, and transcriptional terminator in genomic plasmid (5'→3')

T7 RNA polymerase transcriptional promoter：TAATACGACTCACTATAGGG;

Hepatitis delta virus ribozyme：GGGTCGGCATGGCATCTCCACCTCCTCGCGGT CCGACCTGGGCATCCGAAGGAGGACGCACGTCCACTCGGATGGCTAAGGGAGGGCG;

T7 RNA polymerase transcriptional terminator：CTAGCATAACCCCTTGGGGCCTC TAAACGGGTCTTGAGGGGTTTTTTG.
